# Timing of administration of indocyanine green for fluorescence-guided surgery in pancreatic cancer: response to Shirakawa et al

**DOI:** 10.1186/s12893-020-00881-x

**Published:** 2020-10-07

**Authors:** Peter L. Labib

**Affiliations:** Department of hepatopancreaticobiliary surgery, Derriford Hospital, Derriford Road, Plymouth, PL6 8DH UK

**Keywords:** Pancreatic cancer, Fluorescence guided surgery, Indocyanine green, ICG, Staging laparoscopy, Pancreaticoduodenectomy, Metastasis

## Main text

Dear editors,

I read with interest the recent publication of a study protocol for using near-infrared fluorescence imaging with indocyanine green (ICG) during staging laparoscopy for pancreatic cancer (Shirakawa et al) [1]. As the authors state, staging laparoscopy reduces the probability of finding metastatic disease on subsequent laparotomy, but the risk is still 18% [2]. To prevent unnecessary laparotomies on patients with unresectable pancreatic cancer, trials investigating adjuncts for staging laparoscopy to improve detection of metastatic disease are warranted and welcome.

Previous results on the use of ICG in pancreatic cancer for fluorescence-guided surgery have been conflicting based on the anatomical site of pancreatic cancer being assessed and the timing of ICG administration. ICG is rapidly taken up by hepatocytes and is excreted into the biliary system without re-entering the hepatobiliary circulation [3]. Its ability to highlight liver metastases relies on clearance of ICG from the normal liver parenchyma whilst having impaired clearance from hepatic tumour sites, providing a meaningful tumour-to-background ratio (TBR) detectable to the surgeon. To allow time for ICG clearance, ICG needs to be administered at least 24 h prior to staging laparoscopy (and potentially longer if the patient has any evidence of biliary obstruction). Near-infrared imaging of the primary tumour using ICG has also shown varying results due to timing of administration. Newton and colleagues published two studies (*n* = 4 and *n* = 12) demonstrating that after preoperative (24 h) ICG administration, TBRs of 4.6–4.9 could be achieved in the primary tumour site [4, 5]. However, a study by Hutteman et al. failed to generate meaningful TBRs with intraoperative ICG administration due to physiological uptake in the normal pancreas (likely related to its high blood supply compared to the tumour mass). Regarding peritoneal deposits, studies suggest that ICG clearance is quite rapid. The paper referenced by the authors by Liberale and colleagues describes the administration of intraoperative ICG for the identification of colorectal peritoneal metastases [6]. Their protocol was changed from preoperative (24 h) ICG administration to intraoperative ICG administration after the first patient as no near-infrared signal was detected at laparoscopy 24 h post-administration. Supporting this is their exclusion from further analysis of nodules resected from patients more than six hours post-ICG injection as the fluorescent signal had dissipated. Handgraaf and colleagues published a prospective study combining laparoscopic ultrasound and preoperative (24–48 h) ICG administration in staging laparoscopy for pancreatic cancer(*n* = 25) [7]. Four patients had metastatic disease, two of which were identified by ICG. In the two false negatives, one hepatic metastasis was identified deep to the liver surface by ultrasound and the fourth patient had peritoneal deposits that did not fluoresce.

The problem with the current study protocol is that the primary outcome is trying to simultaneously identify two types of metastatic deposit (hepatic and peritoneal) that have different ideal times for ICG administration. The current protocol for administering ICG one day before staging laparoscopy is ideally suited to the detection of hepatic metastases and pancreatic cancer at the primary site. However, it is unlikely to detect peritoneal deposits as the ICG will have dissipated by this time point. Intraoperative ICG is more likely to detect peritoneal deposits based on the study by Liberale, but this will result in high physiological fluorescence from the liver making identification of small hepatic metastases impossible. One suggested solution is to administer a first bolus of ICG 24 h preoperatively to assess for hepatic metastases after white light imaging as per the current protocol, but to then administer a second bolus of ICG intraoperatively to detect peritoneal metastases. This amendment may identify more metastatic disease than the current protocol, which may ultimately have a significant effect on the study’s primary and secondary outcomes.

I wish the authors good luck in their study and look forward to seeing the results in due course.

## Data Availability

Not applicable.

## Abbreviations

ICG: Indocyanine green
TBR: Tumour-to-background ratio
